# Implementation of Quantum Algorithms via Fast Three-Rydberg-Atom CCZ Gates

**DOI:** 10.3390/e24101371

**Published:** 2022-09-27

**Authors:** Shiqing Tang, Chong Yang, Dongxiao Li, Xiaoqiang Shao

**Affiliations:** 1 College of Physics and Electronic Engineering, Hengyang Normal University, Hengyang 421002, China; 2 College of Physics Science and Technology, Shenyang Normal University, Shenyang 110034, China; 3 Center for Quantum Sciences and School of Physics, Northeast Normal University, Changchun 130024, China; 4 Center for Advanced Optoelectronic Functional Materials Research, and Key Laboratory for UV Light-Emitting Materials and Technology of Ministry of Education, Northeast Normal University, Changchun 130024, China

**Keywords:** multiqubit controlled gate, Rydberg blockade, quantum algorithm

## Abstract

Multiqubit CCZ gates form one of the building blocks of quantum algorithms and have been involved in achieving many theoretical and experimental triumphs. Designing a simple and efficient multiqubit gate for quantum algorithms is still by no means trivial as the number of qubits increases. Here, by virtue of the Rydberg blockade effect, we propose a scheme to rapidly implement a three-Rydberg-atom CCZ gate via a single Rydberg pulse, and successfully apply the gate to realize the three-qubit refined Deutsch–Jozsa algorithm and three-qubit Grover search. The logical states of the three-qubit gate are encoded to the same ground states to avoid an adverse effect of the atomic spontaneous emission. Furthermore, there is no requirement for individual addressing of atoms in our protocol.

## 1. Introduction

Fault-tolerant quantum computing [1], exploiting quantum mechanical phenomena such as superposition and entanglement, is crucial for solving difficult problems in many-body quantum mechanics and mathematics which lack efficient algorithms on classical computers. It also holds a promise for simulation of quantum systems [2], chemistry [3], materials science [4], finance [5], and so on [6,7,8,9,10,11,12]. Any quantum computation can comprise of a sequence of one- and two-qubit quantum gates [13]. Therefore, extensive efforts have been made to achieve high-speed, high-fidelity, and robust two-qubit gates, and various two-qubit gate schemes have been proposed, such as adiabatic gates [14], diabatic gates [15], resonator-induced gates [16], and microwave gates [17].

With the rapid development of quantum information science, an enormous amount of ingenious work, e.g., quantum error correction [18] and quantum algorithms [19], requires the large-scale quantum computation based on the multiqubit controlled operations (the number of qubit is greater than 2). Although the multiqubit controlled operations can be decomposed into a series of universal single- and two-qubit gates, the quantum system becomes more and more complicated as the number of qubits increases, and it is more difficult to get an error per gate (the difference between 1 and average gate fidelity) below the fault-tolerant threshold. Thus, the direct implementation of multiqubit logic gates can greatly simplify the quantum circuit and improve the efficiency and quality of quantum information processing, which results in the increased attention to straight multiqubit gates [20,21,22,23]. For instance, three-qubit controlled gates, a typical class of multiqubit gates, have been demonstrated in many experimental platforms, such as trapped ions [24], Rydberg atoms [25,26], superconducting systems [27], nuclear magnetic resonance [28], and photonic architecture [29]. However, it is still a challenge for the direct realization of a fast multiqubit logic gate with fewer driven fields and without individual addressing of atoms.

Rydberg atoms have long been deemed as a promising platform because of the strong and tunable interactions between Rydberg states, and play important roles in entanglement generation [30,31], quantum simulators [32], quantum state transfer [33], and so on. The interaction can lead to the phenomenon of Rydberg blockade [34], which prevents nearby atoms from being excited to the Rydberg state simultaneously via a single Rydberg excitation. Motivated by the first Rydberg-blockade proposal to perform fast gate operations [35], extensive efforts have been made to improve the gate fidelity and design alternative proposals [36,37,38,39], as well as generalize relevant ideas to multiqubit gates [40,41,42,43,44,45,46]. Particularly, Han et al. [37] implemented fast two-qubit entangling gates via Rydberg blockade and required neither individual addressing of atoms nor adiabatic procedures. Nevertheless, their scheme can not rapidly implement a three-qubit gate, and the logical states have to be encoded by different ground and excited states, which increases the complexity and the adverse effect of the decoherence. Jandura et al. [46] also designed the controlled-Z gate and its three qubit generalization. However, the complicated laser pulses dependent on operation time lead to more restrictions.

In this work, we put forward an alternative scheme to rapidly implement a three-Rydberg-atom CCZ gate via a single Rydberg pulse, where the average gate fidelity can be above 97%. Our protocol not only requires neither individual addressing of atoms nor adiabatic procedures, but also encodes the logical states with the same ground states. In addition, compared with the schemes comprised of step-by-step operations on different atoms [23], our scheme is the one-step implementation scheme. It significantly reduces the complexity of experimental operations and raises the feasibility of experiments. Finally, we successfully apply our proposal to realize three-qubit refined Deutsch–Jozsa algorithm [47,48] and three-qubit Grover search [49,50].

## 2. Principle of the Fast Three-Rydberg-Atom CCZ Gate

**Figure 1:**
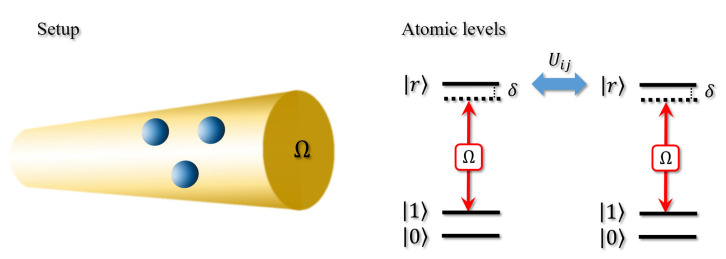
Schematic illustrations for the setup and atomic levels of the three-Rydberg-atom system. Each atom includes two ground states |0,1〉 and one Rydberg state |r〉. The Rydberg state is dispersively coupled to the ground states via one common Rydberg pulse with effective Rabi frequency Ω≈2π×3.5 MHz and adjustable detuning δ. The Rydberg–Rydberg interaction between the *i*- and *j*-th atoms is described as $U_{ij}$.

The system to realize the fast CCZ gate consists of three ${}^{87}$Rb Rydberg atoms trapped in three tweezers with separation $r_{ij}$ shorter than the blocking radius, where $r_{ij}$ is the atomic distance between the *i*- and *j*-th atoms, and there is no requirement for the shape of the atomic arrangement. Each atom includes two ground states and one Rydberg state. The ground states are $|0\rangle \equiv |F=1,m_{F}=0\rangle$ and $|1\rangle \equiv |F=2,m_{F}=0\rangle$ of $5S_{1/2}$ hyperfine clock states with splitting 2π×6.83 GHz, which are used as encoded quantum bits to effective restrain the adverse effect of atomic spontaneous emission. The Rydberg state $|r\rangle \equiv |70S_{1/2},m_{j}=-1/2\rangle$ can be dispersively coupled to the ground states via one common Rydberg pulse [23] with effective Rabi frequency Ω≈2π×3.5 MHz and adjustable detuning δ. Due to the above design, our scheme is simple for experiment and needn’t individual addressing of atoms. The corresponding schematic illustrations for the setup and atomic levels of the three-Rydberg-atom system is shown in Figure 1.

In the interaction picture, the total Hamiltonian can be written as

(1) $$\begin{array}{c} H_{I}=\frac{\Omega }{2}\sum_{j=1}^{3}\sigma_{j}^{r1}e^{i\delta t}+H.c.+\sum_{k>j}U_{jk}\sigma_{j}^{rr}\sigma_{k}^{rr}, \end{array}$$

where ${|\alpha \rangle }_{jj}\langle \beta |$ is parameterized as $\sigma_{j}^{\alpha \beta }$ (α,β=0,1,r) and $U_{ij}$ denotes the Rydberg–Rydberg interaction of *i*- and *j*-th atoms. Here, we consider the Rydberg–Rydberg interactions are caused by the long-range van der Waals interaction, which is equal to $-C_{6}/r_{ij}^{6}$ and $C_{6}=-2\pi \times 862.69$ GHz·μm${}^{6}$ for the Rydberg state $|r\rangle \equiv |70S_{1/2},m_{j}=-1/2\rangle$ [51]. It is noteworthy that the result of our scheme is independent of the functional form of $U_{ij}$ and only the strength of the interaction at a given fixed distance of the two atoms is relevant. Thus, a dipole-dipole interaction is also valid for our scheme.

While the condition of Rydberg blockade $\min(U_{ij})\gg \Omega$ is satisfied, the simultaneous excitations of Rydberg atoms will be suppressed and the Equation (1) can be simplified as an effective Hamiltonian (see Appendix A for details),

(2) $$\begin{array}{c} H_{\mathrm{eff}}=\frac{\Omega }{2}\sum_{j}P_{j-1}^{0}\sigma_{j}^{r1}P_{j+1}^{0}e^{i\delta t}+H.c. \end{array}$$

Here, $P_{j}^{0}=1-\sigma_{j}^{rr}$ and periodic boundary conditions of *j* is considered. Based on the effective Hamiltonian, we can obtain the gate operation in the subspace spanned by {|000〉,|001〉,|010〉,|011〉,|100〉,|101〉,|110〉,|111〉} as

(3) $$\begin{array}{c} C_{s}\left(t\right)=\mathrm{diag}\left\{C_{0},C_{1},C_{1},C_{2},C_{1},C_{2},C_{2},C_{3}\right\}, \end{array}$$

where

$$\begin{array}{ccc} C_{l} & = & \cos\left(\frac{1}{2}\delta \chi_{l}t\right)+i\frac{1}{\chi_{l}}\sin\left(\frac{1}{2}\delta \chi_{l}t\right),\\ \chi_{l} & = & \sqrt{\frac{l\Omega^{2}}{\delta^{2}}+1},\;\;l=0,1,2,3. \end{array}$$

Once a suitable time for the gate operation is selected, we can obtain a gate operation diag{1,1,1,−1,1,−1,−1,1}. To get the target CCZ gate $C_{z}=\mathrm{diag}\{-1,1,1,1,$1,1,1,1}, three single qubit logical gates (operation $\sigma_{j}^{11}-\sigma_{j}^{00}$ for the *j*-th atom) operated on the subspace {|0〉,|1〉} will be further performed in succession, and the Equation (3) can be obtained as

(4) $$\begin{array}{c} C\left(t\right)=\mathrm{diag}\left\{-C_{0},C_{1},C_{1},-C_{2},C_{1},-C_{2},-C_{2},C_{3}\right\}. \end{array}$$

Then we characterize the quality of the gate operation via the trace-preserving-quantum-operator-based average gate fidelity [52,53]

(5) $$\begin{array}{c} \bar{F}\left(\varepsilon ,\hat{U}\right)=\frac{\sum_{\alpha }\mathrm{tr}\left(\hat{U}{\hat{U}}_{\alpha }^{†}{\hat{U}}^{†}\varepsilon \left({\hat{U}}_{\alpha }\right)\right)+d^{2}}{d^{2}\left(d+1\right)}, \end{array}$$

where ${\hat{U}}_{\alpha }$ is the tensor of Pauli matrices III,IIX,…,ZZZ, d=8 is the dimension for the three-qubit logic gate, $\hat{U}$ is the perfect $C_{Z}$ gate, and ε is the trace-preserving quantum operation obtained through our real logic gate C(t), *i.e.,* $\varepsilon \left({\hat{U}}_{\alpha }\right)=C\left(t\right){\hat{U}}_{\alpha }C^{†}\left(t\right)$. The analysis formula of the average gate fidelity for our scheme can be described as

(6) $$\begin{array}{c} \bar{F}=\frac{1}{72}{\left|C_{0}+3C_{1}-3C_{2}+C_{3}\right|}^{2}+\frac{1}{9}. \end{array}$$

Then one can regulate the detuning δ and the operation time to make $\bar{F}$ tend to 1.

**Figure 2:**
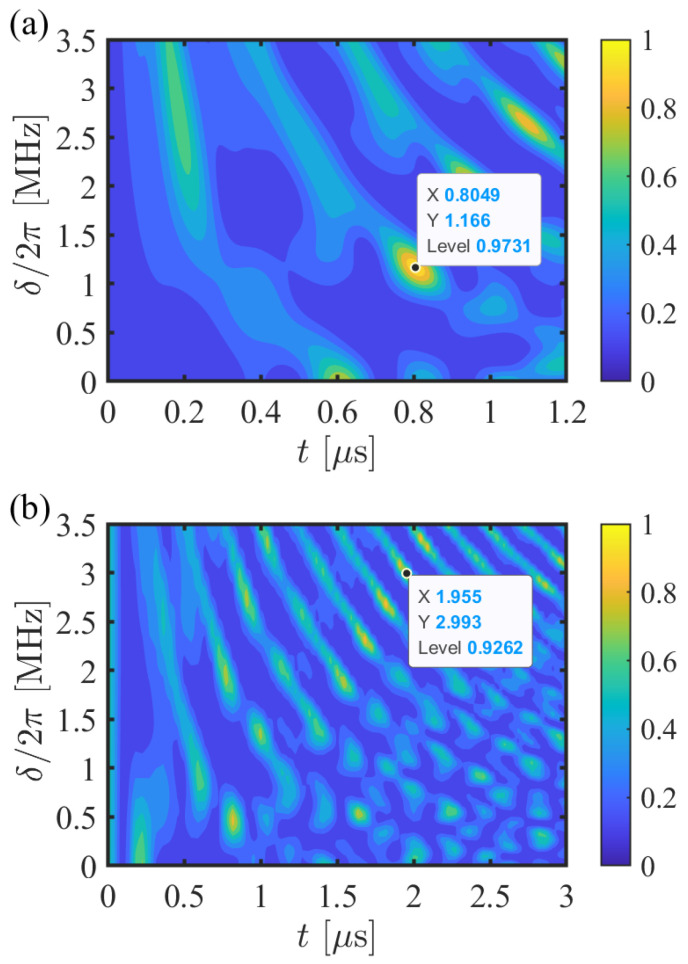
(**a**) The average gate fidelity with respect to δ as well as the gate operation time, where the system is governed by the full Hamiltonian of Equation (1) to realize the gate of Equation (4). (**b**) The average gate fidelity without the three single qubit logical gates as functions of δ and *t*. For the two sub-pictures, the Rabi frequencies and the interaction strengths are Ω=2π×3.5 MHz and $U_{ij}=U\approx 2\pi \times 35$ MHz corresponding the atomic distance $r_{ij}=r\approx 5.4$ μm.

In Figure 2a, we plot the average gate fidelity with respect to δ as well as the gate operation time, where the system is governed by the full Hamiltonian of Equation (1). The average gate fidelity can rapidly reach 97.31% with the gate operation time t=0.8049 μs and δ/2π=1.166 MHz, which is good enough for the direct implementation of the three-qubit CCZ gate. For the other values of δ, it can also be above 90% within t=1.2 μs. These results adequately demonstrate the feasibility and the high efficiency of our scheme. In addition, the CCZ gate can be realized directly without the three single qubit logical gates, i.e., set a suitable time to make $C_{s}\left(t\right)$ equal to the $C_{Z}$ gate. In Figure 2b, we also illustrate the average gate fidelity without the three single qubit logical gates as functions of δ and *t*. Compared with our original scheme, the scheme without the three single qubit logical gates will spend too much time. Furthermore, the average gate fidelity can only arrive at 92.62% with t=1.955 μs and δ/2π=2.993 MHz. The relevant data exhibits the importance for the operation of three single qubit logical gates.

Compared with the implementation of the fast two-qubit entangling gates via Rydberg blockade [37], it is observed that the aim of our scheme is to make the direct implementation of the CCZ gate easier and more efficient. Therefore, our scheme is designed for the three-Rydberg-atom system and cannot be generalized, which is a limitation for the present method. Besides, the method is sensitive to the variations of gate operation time. It is also the fundamental limitation for the schemes governed by the unitary dynamics.

## 3. Applications of Quantum Algorithms

### 3.1. Refined Deutsch–Jozsa Algorithm

Quantum algorithms play an important role in improving computational speed over their classical counterparts due to computational parallelism or interference effects. For the numerous quantum algorithms, the original Deutsch–Jozsa (DJ) algorithm [54] or its modified version (refined DJ algorithm) [47] represents a paradigmatic example, which has been implemented in various systems [55,56,57].

The heart of the original DJ algorithm [54] is to distinguish constant functions $f_{C}\left(x\right)$ from balanced functions $f_{B}\left(x\right)$ in an *N*-qubit system in terms of one query of binary-valued function $f\left(x\right):{\left\{0,1\right\}}^{N}\to \left\{0,1\right\}$. The function can be described as the unitary operation

(7) $$\begin{array}{c} U_{f}|x\rangle |y\rangle =|x\rangle |y⊕f\left(x\right)\rangle , \end{array}$$

where *x* is an *N*-qubit input and *y* is the auxiliary qubit. To improve the original DJ algorithm, Collins et al. [47] proposed the refined DJ algorithm that fully removes the auxiliary qubit *y*. The corresponding action of the *f*-controlled gate can be expressed as [47,48]

(8) $$\begin{array}{c} U_{f}^{N}|x\rangle ={\left(-1\right)}^{f(x)}|x\rangle . \end{array}$$

**Figure 3:**
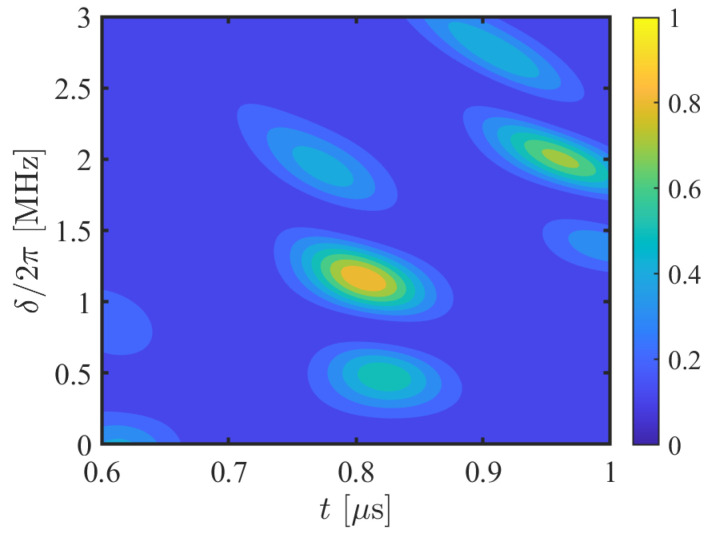
Contour plot of average gate fidelity of $U_{B1}^{3}$ with respect to the detuning and the gate operation time. The relevant parameters are the same as those of Figure 2a.

For the three-qubit system N=3, there are one *f*-controlled gate of the constant functions $U_{f_{C}}^{3}=\mathrm{diag}\left\{1,1,1,1,1,1,1,1\right\}$ and 35 nontrivial and distinct *f*-controlled gates of the balanced functions $U_{f_{Bj}}^{3}$(j=1,2,…,35). Here, $U_{f_{Bj}}^{3}$ can be decomposed into the combination of CCZ gate $J_{111}=\mathrm{diag}\left\{1,1,1,1,1,1,1,-1\right\}$ and single qubit logical gates of the *k*-th atom $\sigma_{x,k}=\sigma_{k}^{10}+\sigma_{k}^{01}$ [37]. For example, the *f*-controlled gate $U_{B1}^{3}=\mathrm{diag}\left\{1,-1,1,-1,-1,1,1,-1\right\}$ can be constituted as

(9) $$\begin{array}{c} U_{B1}^{3}=J_{111}J_{100}J_{011}J_{001} \end{array}$$

with $J_{100}=\sigma_{x,3}\sigma_{x,2}J_{111}\sigma_{x,2}\sigma_{x,3}$, $J_{011}=\sigma_{x,1}J_{111}\sigma_{x,1}$, and $J_{001}=\sigma_{x,2}\sigma_{x,1}J_{111}\sigma_{x,1}\sigma_{x,2}$. As for the gate $J_{111}$, it can be implemented by replacing the dispersive coupling |1〉↔|r〉 with the dispersive coupling |0〉↔|r〉 in our three-qubit CCZ gate scheme. In Figure 3, we illustrate the contour of average gate fidelity of $U_{B1}^{3}$ with respect to the detuning and the gate operation time. The three-qubit Rydberg system is also governed by the original Hamiltonian. It can be found that the *f*-controlled gate of the balanced function can be achieved with a high fidelity 89.84% as t=0.8049 μs and δ/2π=1.166 MHz, which certifies the feasibility of the application.

### 3.2. Grover Search

Grover search [49] is another remarkable quantum algorithm to find out a certain state and is widely used in various fields [58,59,60,61,62,63,64], which can be carried out via three steps [65]. Firstly, one can employ Hadamard gates to prepare a superposition state $|\psi_{0}\rangle =\sum_{\alpha =0}^{N-1}|\alpha \rangle /\sqrt{d}$, where *d* is the dimension of the system. The second step is to perform an iteration *Q* including two operations: (a) Take advantage of quantum phase gate $I_{\tau }=I-2|\tau \rangle \langle \tau |$ (*I* is the identity matrix) to get the inversion of the amplitude of the marked state |τ〉; (b) Use the diffusion transform *D* ($D_{\alpha \beta }=2/d-\delta_{\alpha \beta }$, α,β=1,2,…,d) to get the inversion about the average of the amplitudes of all states. Finally, the marked state can be obtained by a measurement of the whole system. In this section, we discuss the application of our scheme on the three-qubit Grover search.

For our three-qubit system, the Hadamard gate can be defined as

$$H^{⊗3}={\left(\frac{1}{\sqrt{2}}\right)}^{3}\left(\begin{array}{cc} 1 & 1\\ 1 & -1 \end{array}\right)⊗\left(\begin{array}{cc} 1 & 1\\ 1 & -1 \end{array}\right)⊗\left(\begin{array}{cc} 1 & 1\\ 1 & -1 \end{array}\right),$$

which can be performed via external microwave pulses. The iteration *Q* for the second step can be characterized as

(10) $$\begin{array}{c} Q=H^{⊗3}C\left(t\right)H^{⊗3}I_{\tau }, \end{array}$$

where the C(t) can be obtained by the original Hamiltonian of Equation (1). While the gate operation time *t* is suitable, we can obtain an approximate three-qubit quantum phase gate with

(11) $$\begin{array}{c} C(t)\approx \mathrm{diag}\{-1,1,1,1,1,1,1,1\}. \end{array}$$

Then a full Grover search for three qubits is available through our scheme.

**Figure 4:**
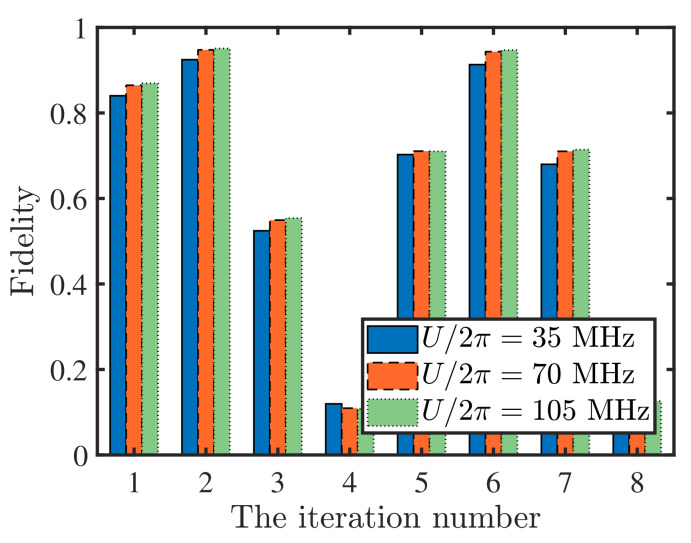
Fidelity of the state searched for as functions of the iteration number with different Rydberg–Rydberg interaction strength. The marked state is |101〉 and the initial state is |000〉. The relevant parameters are the same as those of Figure 2a and the gate operation time for C(t) is set as 0.8049 μs. For simplicity, the Rydberg–Rydberg interaction strength between the *i*- and *j*-th atom are assumed as $U_{ij}=U$.

In Figure 4, we take the marked state |101〉 as an example and calculate the fidelity of the state searched for as functions of the iteration number with different Rydberg–Rydberg interaction strength. For simplicity, the Rydberg–Rydberg interaction strength between the *i*- and *j*-th atom has been assumed as $U_{ij}=U$. The result is good enough for the three-qubit Grover search with the second iteration and U/2π=35 MHz, where a fidelity of up to 92.46% can be acquired. Moreover, the fidelity can be also improved to 94.76% and 95.08% with the increasing of the Rydberg–Rydberg interaction strength. These can fully reflect the feasibility for the application of our scheme to the three-qubit Grover search.

## 4. Discussion and Summary

While the ambient temperature is chosen as {0,77,300,700}K, the effective lifetime of 70$S_{1/2}$ for Rb atoms will be {410.41,287.78,151.55,92.257}μs, respectively [66]. Consequently, the operation time of all the above schemes that can be achieved within 1 μs is much shorter than the effective lifetime of the Rydberg state.

In summary, we successfully achieve a fast three-Rydberg-atom CCZ gate via a common Rydberg pulse and apply it to the three-qubit refined Deutsch–Jozsa algorithm and three-qubit Grover search. In our scheme, the Rydberg blockade effect is used to inhibit the simultaneous excitations of Rydberg atoms. The logical states are encoded into the same ground states to avoid the adverse effect of the atomic spontaneous emission. Additionally, compared with the previous scheme, our proposal requires neither individual addressing of atoms nor step-by-step operations on different atoms. Accordingly, the complexity of experimental operations is reduced and the feasibility of experiments is raised significantly. With the current experimental technologies, the average gate fidelity of the three-Rydberg-atom CCZ gate can be above 97% with a short operation time. We believe the present scheme supplies a viable prospect for the realizations of multiqubit gate and quantum algorithms.

## Data Availability

Data available on request from the corresponding author.

## Appendix A

Here, we will show the detailed derivation from the Equation (1) to the Equation (2) of the main text. For simplicity, we can choose the interaction strengths $U_{12}=U_{13}=U_{23}=U$, and the Equation (1) can be rewritten as

(A1) $$\begin{array}{c} H_{I}=\frac{\Omega }{2}\sum_{j=1}^{3}\sigma_{j}^{r1}e^{i\delta t}+H.c.+U\sum_{k>j}\sigma_{j}^{rr}\sigma_{k}^{rr}. \end{array}$$

Then we utilize the formula $i{\dot{U}}_{0}^{†}U_{0}+U_{0}^{†}H_{I}U_{0}$ to reformulate the Hamiltonian in a rotating frame with respect to $U_{0}=\exp\left\{-itU\sum_{k>j}\sigma_{j}^{rr}\sigma_{k}^{kk}\right\}$ as [67,68],

(A2) $$\begin{array}{c} H_{U}=\frac{\Omega }{2}\sum_{j,m,n}P_{j-1}^{m}\sigma_{j}^{r1}P_{j+1}^{n}e^{i[(m+n)U+\delta ]t}+H.c., \end{array}$$

where m,n=0,1, $P_{j}^{0}=1-\sigma_{j}^{rr}$, $P_{j}^{1}=\sigma_{j}^{rr}$, and periodic boundary conditions of *j* is considered. While the condition of Rydberg blockade U≫Ω is satisfied, the simultaneous excitations of Rydberg atoms will be suppressed and the Equation (A2) can be simplified as the effective Hamiltonian, i.e., the Equation (2) of the main text,

(A3) $$\begin{array}{c} H_{\mathrm{eff}}=\frac{\Omega }{2}\sum_{j}P_{j-1}^{0}\sigma_{j}^{r1}P_{j+1}^{0}e^{i\delta t}+H.c. \end{array}$$
